# Roasting and Cacao Origin Affect the Formation of Volatile Organic Sulfur Compounds in 100% Chocolate

**DOI:** 10.3390/molecules28073038

**Published:** 2023-03-29

**Authors:** Aaron M. Wiedemer, Alan P. McClure, Erich Leitner, Helene Hopfer

**Affiliations:** 1 Department of Food Science, The Pennsylvania State University, University Park, PA 16802, USA; wiedemer.7@buckeyemail.osu.edu; 2 Patric Food and Beverage Development, Columbia, MO 65202, USA; alan@patricdevelopment.com; 3 Institute of Analytical Chemistry and Food Chemistry, Graz University of Technology, 8010 Graz, Austria; erich.leitner@tugraz.at

**Keywords:** chocolate, volatile organic sulfur compounds, comprehensive gas chromatography, roasting, cacao origin

## Abstract

Chocolate is a highly appreciated food that develops its characteristic flavors in large part during the roasting of cacao beans. Many functional classes have been noted for their importance to chocolate flavor, including volatile organic sulfur compounds (VSCs). Despite this, the effect of roasting on the concentration of VSCs has never been thoroughly assessed. Here, we studied the effects of roasting temperature, time, and cacao origin on the formation of VSCs. Twenty-seven 100% chocolate samples made from cacao from three different origins and roasted according to an I-optimal experimental design were analyzed by comprehensive gas chromatography with sulfur-selective detection (GCxGC-SCD). For two compounds, dimethyl disulfide and dimethyl trisulfide, the effects of roasting time, roasting temperature, and cacao origin were modelled using response surface methodology and semi-quantified relative concentration. Overall, roasting increased the number of sulfur-containing volatiles present in chocolate, with a total of 28 detected, far more than previously thought. Increased roasting time and especially roasting temperature were found to significantly increase the concentration of VSCs (*p* < 0.05), while cacao origin effects were only seen for dimethyl disulfide (*p* < 0.05). The identity of most VSCs remains tentative, and more research is needed to unravel the impact of these volatiles on flavor perception in chocolate.

## 1. Introduction

Chocolate is a usually sweetened, solid paste made from cocoa beans with a unique taste, texture, and aroma that make it a highly popular and widely enjoyed food. Chocolate is made from the processed seeds (cacao beans) of *Theobroma cacao*, a small, tropical evergreen tree in the *Malvaceae* family [1]. Cocoa beans are an important global commodity and are mostly grown by small-scale farmers living within 15–20° North and South of the equator [2]. After being harvested, cocoa beans undergo a 5–7-day fermentation process, which is important for flavor precursor development. After fermentation, beans are dried to around 7% moisture content and then transported to chocolate manufacturers [3]. Upon arrival, they are cleaned, deshelled, roasted, ground into a paste so-called cocoa liquor, refined, potentially mixed with other ingredients (e.g., sugar, milk, cocoa butter, flavorings, stabilizers), conched, and then tempered and molded or enrobed as finished chocolate products [3].

Chocolate’s unique flavor is due to a complex mix of both volatile aroma compounds and non-volatile compounds held within a matrix of cocoa fat [4]. The aroma fraction is one of the main contributors to flavor in chocolate, and from a chemical standpoint, it is extremely complex. Thus far, over 600 volatile compounds have been identified in roasted chocolate [4,5,6,7,8]. Besides chocolate volatiles, other constituents such as non-volatile polyphenols, methylxanthines, and non-volatile acids contribute the basic taste and mouthfeel qualities of bitterness, astringency, and sourness, and the ~50% fat (cocoa butter) plays an important role in melting properties and in-mouth flavor release.

The chocolate aroma fraction results both from the genetic variety in cacao and its lengthy and labor-intensive processing [3,4,6]. Of particular importance to the development of chocolate aroma is the roasting step, in which numerous Maillard and Strecker degradation reactions convert flavor precursors produced by fermentation and drying into the main classes of key components, such as pyrazines, aldehydes, and other heteroatom-containing aroma compounds. A recent report by McClure et al. (2022) revealed the effects of roasting temperature and time on chocolate flavor and acceptability and found that, in general, roasting led to decreased perception of bitterness and astringency intensity and increased perceived chocolate aroma intensity as well as overall liking [9].

Sulfur compounds have been widely accepted as being important to the flavor of food due to their low detection thresholds [10,11]. Volatile organic sulfur compounds (VSCs), especially dimethyl disulfide (DMDS) and dimethyl trisulfide (DMTS), have been noted for their importance to chocolate flavor in earlier work [8,12,13,14,15]. These sulfur compounds are thought to result from Strecker degradation of sulfur-containing amino acids and peptides, such as methionine and cysteine, and reducing sugars present in the cocoa bean [15,16,17]. Despite the importance of these compounds stressed in prior literature, the effect of roasting on changes in VSCs has never been thoroughly studied [4,15], potentially due to the analytical challenge associated with the high reactivity and low concentration levels of VSCs in foods [10,11].

The aim of this study was to determine the effects of roasting temperature, roasting time, and cacao origin on the formation of volatile organic sulfur compounds in 100% chocolate samples. We hypothesize that VSC concentration will increase with increasing roasting time and temperature and that VSC concentration will differ across different cacao origins. The objectives of this study were to

1. characterize VSCs in different roasting treatments of 100% dark chocolate from three different origins (Ghana, Peru, and Madagascar) using comprehensive gas chromatography paired with sulfur-selective chemiluminescence detection (GCxGC-SCD);
2. determine the effect of roasting time, roasting temperature, and cacao origin on the concentration of VSCs in 100% dark chocolate;
3. model the effects of roasting time, roasting temperature, and cacao origin on the concentration of VSCs of noted importance to cacao flavor, dimethyl disulfide (DMDS) and dimethyl trisulfide (DMTS).

## 2. Results

### 2.1. The Roasting of Chocolate Increases Number and Concentration Levels of VSCs

**Figure 1:**
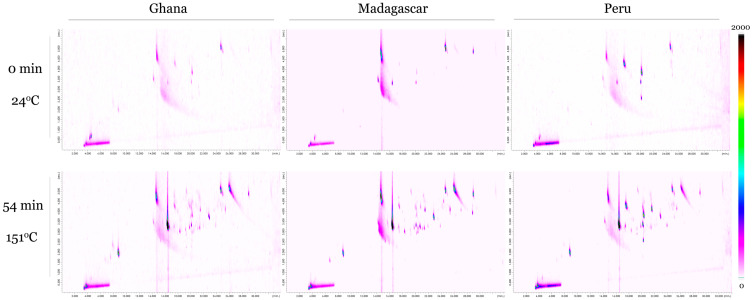
Comprehensive GCxGC-SCD chromatograms for (**top**) unroasted (0 min, 24 °C) and (**bottom**) highly roasted (54 min, 151 °C) chocolate samples for the three cacao origins (Ghana, Madagascar, Peru). All chromatograms are scaled the same to allow for direct comparison (0 to 2000 a.u.).

**Figure 1** provides a visual comparison of comprehensive GCxGC-SCD chromatograms between unroasted (0 min, 24 °C) and highly roasted (54 min, 151 °C) chocolate samples across the three origins (Ghana, Peru, and Madagascar). Each chromatogram is scaled the same, with individual VSCs appearing as peak circles in the chromatogram and increasing in red/brown color with increasing concentration.

Compared to the unroasted samples, the highly roasted chocolate samples show both higher concentrations and a greater number of VSCs. This can be seen in the samples roasted at 151 °C for 54 min having much darker and more VSC peaks than the raw samples. It is also apparent that there are VSCs already present in raw chocolate, albeit at a lower concentration. This trend holds true across all three cacao origins, although VSC concentration levels appear to be higher in the Madagascar samples compared to the Peruvian and Ghanaian chocolate samples.

Looking at individual VSCs, we were able to detect and semi-quantitate 28 volatile sulfur compounds across all chocolate samples, listed in **Table 1** with their average retention indices (RIs) on the first dimension and their average retention times (RTs) for the second dimension. Among the 28 VSCs, we were further able to identify dimethyl disulfide (DMDS; RI_1D_ = 743 ± 1.5) and dimethyl trisulfide (DMTS; RI_1D_ = 976 ± 0.0) by matching them to authentic standards. The remaining compounds are labeled Cpd1 through Cpd26 in order of their average RIs in the first dimension. The chromatographic region with RI_1D_ = 960 ± 25.4 and RT_2D_ = 2.64 ± 0.26, where VSCs showed a strong overlap, could not be separated and was excluded from further analysis.

**Figure 2:**
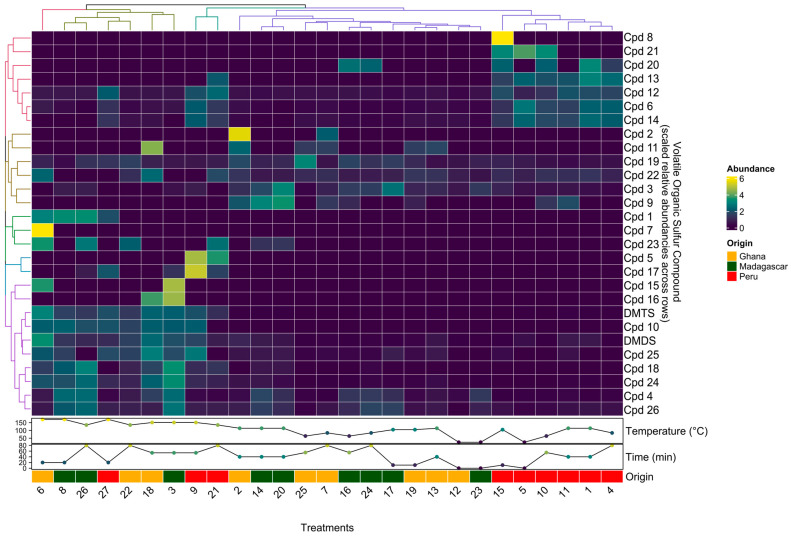
A clustered heatmap of scaled relative concentrations of the 28 VSCs detected in chocolate samples made from beans from three origins (Madagascar, Ghana, and Peru) and roasted at eight different times and temperatures according to an experimental design.

To better compare change patterns across the 28 VSCs detected and the 27 samples (i.e., roasting treatments and origin), root-mean-square scaled relative concentrations were visualized in a clustered heatmap shown in **Figure 2**.

In general, samples roasted at the highest temperatures (>135 °C) showed the highest levels of DMDS, DMTS, Cpd10, Cpd25, and Cpd18. Although not shown here due to scaling, DMTS was the most abundant VSC in all samples roasted >135 °C, increasing dramatically (~4 changes in order of magnitude) once roasting temperatures reached 135 °C. Similar to **Figure 1**, longer roasting times and especially higher roasting temperatures increased both the number and concentration levels of compounds.

Samples from Ghana and Madagascar roasted at cooler temperatures (<114 °C) tended to have the highest levels of Cpd2, Cpd11, Cpd19, Cpd22, Cpd3, and Cpd9.

Interestingly, the VSC composition in Peru samples appears to be more similar across all roasting treatments, as six of the nine Peru samples formed a cluster; these six samples were roasted at low to medium temperatures; this origin also appears to be the most different from the other two origins, Ghana and Madagascar. Peruvian samples roasted at lower temperatures (<114 °C) showed the highest levels of Cpd8, Cpd21, Cpd20, Cpd13, Cpd12, Cpd6, and Cpd14, all of which were below detection levels or at much lower levels in the other two origins.

Lastly, we were able to detect origin-specific VSCs that only appeared or appeared in dramatically higher concentrations in one origin but not the others. For example, compound Cpd4 was present in much higher concentrations in samples from Madagascar, while compound Cpd13 was detected only in samples from Peru.

Although it appears that some VSCs that are detected in samples roasted at lower temperatures below 114 °C have decreased in concentration, once samples are roasted at higher temperatures above 135 °C, this is likely not true and rather a result of our cut-off criterion to only look at the 20 most abundant compounds present in each sample. Due to the dramatic increase in the number of VSCs once roasting temperatures surpass 135 °C, these newly formed VSCs exceed any previously present ones in concentration.

### 2.2. Roasting Time, Roasting Temperature and Cacao Origin Affect DMDS and DMTS Formation in Chocolate

For the two identified VSCs, dimethyl disulfide (DMDS) and dimethyl trisulfide (DMTS), we further studied how the experimental factors (roasting time, roasting temperature, cacao origin) affect the formation of these compounds.

**Figure 3:**
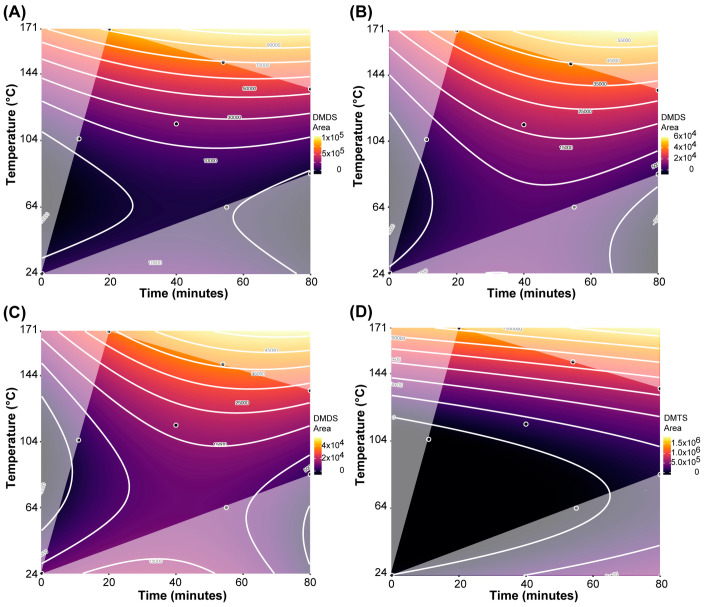
Response surface contour plots for dimethyl disulfide (DMDS) and dimethyl trisulfide (DMTS), as a function of roasting time and roasting temperature. (**A**) DMDS in samples from Ghana; (**B**) DMDS in samples from Madagascar; (**C**) DMDS in samples from Peru; (**D**) DMTS in samples across all three origins.

For dimethyl disulfide (DMDS), the BIC-selected model (adjusted R^2^ = 0.939; **Table 2**) showed that all three factors as well as certain interactions and higher-order terms all significantly affect DMDS concentration in the samples. The corresponding response surface contour plots, depicting the concentration changes in DMDS as a function of roasting time and roasting temperature separated by cacao origins, are shown in **Figure 3A–C**. The relative concentration of DMDS increases primarily due to roasting temperature but is also significantly affected by roasting time and interactions between temperature and cocoa origin. However, even with these significant interaction effects, including cocoa origin, there is still a relatively similar pattern of DMDS increase for each origin. Samples from Ghana showed the greatest increase in DMDS relative concentration, while chocolate from Peru had the smallest changes in DMDS relative concentration due to roasting. This would indicate that DMDS formation due to roasting is similar across the origins, but depending on the availability of precursors, DMDS is formed at different levels. Interestingly, some low levels of DMDS are present in all raw samples.

For dimethyl trisulfide (DMTS), the BIC-selected model indicated that only roasting time and particularly roasting temperature, but not cacao origin, were significant predictors of DMTS in the chocolate samples (**Table 2**). Comparing the model coefficients, the effect of roasting time was less than 1/3 of the effect of roasting temperature. This is also visualized in the response surface contour plot (**Figure 3D**): the relative concentration of DMTS increases primarily due to roasting temperature but is also significantly affected by roasting time. Roasting temperature is particularly effective in increasing DMTS at higher temperatures, apparent by a 355-fold increase in DMTS in the Ghana samples from the raw sample to the samples roasted at 151 °C for 54 min and 171 °C for 20 min.

Comparing the three experimental parameters to each other, the significant quadratic term for roasting temperature for both DMDS and DMTS indicates that roasting temperature appears to be the most important factor in forming VSCs. For both DMTS and DMDS, it was found that concentration levels dramatically increase once a temperature threshold of about 135 °C is surpassed.

## 3. Discussion

Volatile organic sulfur compounds (VSCs) and their changes due to roasting in 100% chocolate from three different cacao origins were characterized for the first time using untargeted comprehensive gas chromatography with sulfur-selective detection.

Results revealed a large magnitude of VSCs in chocolate, many of which were already detected in raw, unroasted chocolate. In total, 28 VSCs, including dimethyl disulfide (DMDS) and dimethyl trisulfide (DMTS), representing the top 20 most abundant VSCs across the 27 samples, were further characterized to determine the effects of cacao origin (Ghana, Peru, and Madagascar) and roasting temperature and time. We could only unambiguously identify two VSCs, DMDS and DMTS, with authentic standards, revealing the need for more research. Inspecting the comprehensive gas chromatograms, many VSCs appear to co-elute along the first dimension, i.e., have identical and/or very similar RIs, further evidencing the analytical challenge posed by the detection and identification of sulfur compounds in complex foods such as chocolate.

The presence of at least 26 other VSCs in chocolate besides DMDS and DMTS further demonstrates the urgent need for future work. Thus far, only DMS, DMDS, DMTS, 3-(methylthio)propanal (=methional), and 2-methyl-3-(methyldithio)furan have been reported in chocolate and cocoa mass [4,5,6,7,8,14,18]. Future work is needed to uncover the chemical identity of these VSCs, given VSCs’ general importance to food flavor [10,11].

The general trend of roasting temperature and time increasing the concentration and number of VSCs in chocolate seen across nearly all detected VSCs supports the proposed formation by Liu and coworkers [15]. VSCs likely form from the Strecker degradation reactions of heated reducing sugars and sulfur-containing amino acids present in cocoa. Protein content in mature cacao beans ranges from 10–16% (dry weight), primarily as proteins, which increase to 1–2% (dry weight) of free amino acids after fermentation [6,19]. Strecker degradation of methionine was found to produce dimethyl sulfide (DMS), DMDS, and DMTS [20]. Earlier work has shown that raw cocoa bean extract, roasted cocoa beans, and cocoa nibs contain the sulfur-containing amino acids methionine and cysteine [17,21,22,23].

All treatments, including the raw, unroasted samples, showed detectable levels of VSCs. This indicates that VSCs could also result from other processing steps, such as fermentation and drying, where beans are exposed to temperatures above 40 °C for several days [3], and/or methionine and other sulfur compounds could be metabolized by various microorganisms to form VSCs [24]. Lopez and Quesnel (1976) report the presence of methyl-S-methionine sulphonium salt in fermented and dried cocoa beans that readily decomposes into dimethyl sulfide [25]. Additional work, e.g., labeling studies, is needed to elucidate the formation of VSCs in chocolate.

Roasting dramatically increased both the number and concentration levels of the 28 VSCs, and this was to some degree also affected by cacao origin, with generally fewer compounds detected in the beans from Peru. Roasting time and particularly roasting temperature increased the concentration levels of almost all VSCs. For DMDS and DMTS, this effect was modeled, and for both DMDS and DMTS, the model coefficients for roasting temperature were at least three times greater than those for roasting time. In addition, for both compounds, the quadratic temperature term also significantly affected concentration levels. These findings support our initial hypothesis that increased roasting temperature and time increase the relative concentration of VSCs.

In this work, we were able to detect some VSCs that did not increase due to roasting (e.g., Cpd20) and/or were detected in some but not all samples (e.g., Cpd2, which is only seen in two samples from Ghana). It is possible that these compounds were present in more samples but were below the concentration cut-off level used to select only the 20 most abundant compounds in every sample. Future analyses should expand the number of VSCs included in the analysis.

Although not a major driver of observed changes, origin was shown to affect some VSCs. Cacao origin was shown to significantly interact with roasting temperature in the regression model for DMDS, while origin alone was not found to significantly affect DMTS concentration. This would suggest that at least for DMDS, formation is dependent on available precursors that appear to vary between origins. For DMTS, origin was not a significant predictor in the regression model. Evidence of an origin effect was also found for some of the other 26 VSCs, where certain compounds only appear in samples from a certain origin (e.g., Cpd13 in chocolates from Peru) and/or certain VSCs are present in higher concentrations in some origins across roasting treatments. For example, the compound Cpd4 is present in much higher concentrations in the Madagascar samples. An origin effect was found for only some VSCs; thus, our hypothesis that VSCs would be affected by cocoa origin was only partially supported. It could be that VSCs vary from other origins not studied here. Thus, more work is needed to elucidate the effect of cacao origin on VSC formation during roasting. Nevertheless, this study is one of the first to systematically assess the effect of various cacao origins on the chemical composition of chocolate while controlling for the impacts of roasting (i.e., time and temperature)—a major strength of this work, as the impact of origin has been frequently overlooked [4,9,26].

Given the importance of volatile sulfur compounds in the flavor of most foods [10,11] and the previously reported importance of DMDS and DMTS in chocolate flavor [4,8,13,14,15] it is very likely that some (or all) of the VSCs detected here may also contribute to chocolate flavor perception. Given that previously reported important aroma molecules in dark chocolate, such as 2-methoxy-3-isopropylpyrazine and 2,3-diethyl-5-methylpyrazine, by themselves do not smell chocolatey [8,14,18,27,28,29], it appears that chocolate aroma is a ‘gestalt’ or a perceptual image of individual compounds [30]. This is further supported by Lopez and Quensel (1974), who report that adding certain VSCs to 3-methylbutanal gives it a chocolate aroma [13].

Results from this study show that roasting plays an essential role in the formation of these compounds. Particularly for DMTS, once roasting temperatures exceed ~135 °C, according to the regression model, concentration levels increase around 350-fold compared to the unroasted sample. This is of particular note, as our earlier work [9] showed that the perception of cocoa flavor and bitter taste intensity were similarly changing with roasting time and temperature. Volatile organic sulfur compounds may be another important compound class in chocolate aroma. For example, dimethyl sulfide has been reported as an important “odor enhancer” in wine [31,32], a similarly complex matrix to chocolate. A similar indirect effect of DMDS on cocoa and chocolate is therefore plausible, especially in light of the reports by Lopez and Quesnel [13].

In conclusion, this study was the first to systematically reveal the effects of roasting and cocoa bean origin on the formation of volatile organic sulfur compounds. The use of advanced comprehensive gas chromatography with sulfur-selective detection (GCxGC-SCD) is a major strength of this work. The use of comprehensive GC revealed the significant co-elution of many VSCs along the first dimension, calling earlier work on important aroma compounds in chocolate to some degree into question. Pairing GCxGC with sulfur-selective detection allowed for an accurate depiction of VSCs in a wide range of possible roasting treatments. The formal experimental design allowed for the systematic characterization of two important VSCs in chocolate: dimethyl disulfide and dimethyl trisulfide.

We found that VSC formation, including DMDS and DMTS, is primarily driven by roasting temperature but is also influenced by bean origin, likely due to different concentrations of and possibly the nature of the precursors. Interestingly, we found that DMDS showed a significant effect of cocoa origin, whereas this was not the case for DMTS. This source of variability and differences in formation between chemically very similar compounds has often been overlooked in previous work. Future work should aim at deciphering this origin effect—is it genetic or environmental in nature, or perhaps both?

This study was exploratory in nature; thus, weaknesses include the use of relative concentrations and the identification of only two VSCs with authentic standards. The applied cut-off level of focusing on the top 20 most abundant VSCs across all samples is another point that future work could improve on, as chromatograms indicate that there are many more VSCs present in the samples. Further, there is the potential for artifact formation due to the use of a hot inlet desorption (vs. e.g., cool-on-column injection), and while several groups report thiol oxidation at increased inlet temperatures [33,34], the presence of oxygenated compounds (e.g., acetic acid) seems to lower such degradation [33]. Cocoa is a complex mixture, where VSCs are present alongside organic acids, carbonyls, and many others [4,35]. Although we cannot exclude potential artifact formation during analysis, we expect such systematic effects to be the same across all samples, thus conserving all relative sample differences in VSC composition. Future work should compare the impact of the analysis method on the VSC composition analysis of cocoa.

The results represent a conservative estimate of volatile organic sulfur compounds in chocolate, so more research is needed to better understand the nature, formation, and impact of VSCs on chocolate aroma.

## 4. Materials and Methods

### 4.1. Chocolate Samples and Chemicals

All chocolate samples were sourced and prepared as described in detail previously [9,26]. Briefly, well fermented, dried cacao beans from three origins (Ghana, Madagascar, and Peru) were roasted according to an I-optimal experimental design (**Table 3**), covering a large region inclusive of a wide range of settings used in the chocolate industry, with roasting temperature ranging from 24 °C to 171 °C and roasting time varying between 0 to 80 min, including a “raw”, unroasted treatment at 24 °C for 0 min. The resulting design space included a total of nine treatments (eight unique temperature-time combinations plus one duplicated center point) for each of the three origins (=27 total chocolate samples). All chemicals required for analysis were obtained from Sigma-Aldrich (Steinheim, Germany) and Fluka GmbH (Buchs, Switzerland), and were of analytical grade or higher in purity.

### 4.2. Volatile Analysis

Approximately 250 g of each chocolate sample was double wrapped in heavy-duty aluminum foil, placed in labelled plastic bags, and transported by air in insulated tote bags from State College, PA, USA, to Graz, Austria, for volatile analysis. Upon arrival, samples were removed from the bags and aluminum foil, broken up into coarse pieces, and stored in 40-mL glass vials at 4 °C in the dark.

#### 4.2.1. Optimization of Headspace-Solid Phase Microextraction (HS-SPME) Parameters

Two center-point samples (14: Madagascar, 40 min, 114 °C; 11: Peru, 40 min, 114 °C) were chosen to optimize the HS-SPME extraction parameters, varying extraction time and temperature (40 °C at 20 min and 40 min, 60 °C and 80 °C at 20 min) and sample weight (50 mg, 100 mg, 250 mg, 500 mg). For time and temperature optimization analyses, cocoa samples (100.0 ± 1.5 mg) were prepared in triplicate in 20 mL HS vials (Shimadzu Europa GmbH, Duisburg, Germany) along with a glass-coated magnetic stir bar and capped with a polytetrafluoroethylene (PTFE)-lined silicone septum magnetic crimp cap. Similarly, to test the effects of different sample weights (50 mg, 100 mg, 250 mg, and 500 mg), samples were prepared for each weight in duplicate (±1.5 mg). All optimization experiments were measured by gas chromatography-flame ionization detection (GC-FID) (see Section 4.2.2). The optimal parameters were found to occur with an extraction temperature of 60 °C for 20 min, as chromatograms showed the number of different compounds and peak areas to stabilize at 60 °C with no noticeable difference between 60 and 80 °C and 20 min being more efficient compared to longer extraction times. For sample weight optimization, no discernible difference between the sample weights was found; therefore, a sample weight of 100 mg was chosen to conserve sample and increase accuracy.

#### 4.2.2. Profiling of Volatile Sulfur Compounds with GCxGC-SCD/FID

All VSCs were analyzed in all chocolate samples in analytical triplicate using comprehensive gas chromatography coupled with sulfur chemiluminescence and flame ionization detection (GCxGC-SCD/FID). For analysis, 100.0 ± 0.5 mg of chocolate samples were weighed into 20-mL HS vials together with a glass-coated magnetic stir bar and enclosed with a polytetrafluoroethylene (PTFE)-lined silicone septum magnetic crimp cap (Shimadzu Europa GmbH). Due to the exploratory nature of this study and the lack of data on VSCs in chocolate, no internal standard was used.

A Shimadzu Nexis 2030 GC with liquid nitrogen cryogenic cooling (Shimadzu Europa GmbH, Duisburg, Germany), coupled with SCD and FID (Shimadzu Europa GmbH), and equipped with a CTC PAL 2 autosampler with a Chromtech single magnetic mixer (CTC Analytics, Zwingen, Switzerland), was used for analysis. Volatiles were automatically extracted for 20 min at 60 °C using a 2 cm DVB/Car/PDMS SPME fiber (Supelco, Bellefonte, PA, USA), after which the fiber was thermally desorbed for 4 min in a SPME inlet liner (Supelco), held at 250 °C in splitless mode (purge valve opened after 1 min).

Separation on the first dimension was achieved on a nonpolar SLB-5MS column (20 m × 0.18 mm inner diameter × 0.18 μm film thickness; Supelco), paired with a mid-polarity column on the second dimension (SLB 35-MS; 5 m × 0.32 mm inner diameter × 0.25 μm film thickness; Supelco). The temperature program started at 25 °C for 1 min and ramped from 5 °C/min to 190 °C for a total run time of 34 min, with helium (99.999% purity, Linde, Graz, Austria) as a carrier gas. Initial column head pressure was set at 102 kPa (1 min), followed by a temperature program of 2.5 kPa/min to 185 kPa. A Trajan flow modulator (Ringwood, Victoria, Australia) was used with a modulation frequency of 6 s and a loop fill time of 400 ms. With an additional flow control module (APC1), the loop was flow-programmed with an initial 61.5 kPa and a pressure program of 120.8 kPa. SCD data was acquired with a sampling rate of 8 ms, a delay time of 0.0 min, and no detector subtraction, with the detector interface temperature set at 200 °C and the SCD furnace temperature held at 850 °C. The detector used a hydrogen flow of 80.0 mL/min, a nitrogen flow of 40.0 mL/min, an oxygen flow of 10.0 mL/min, and an ozone flow of 25.0 mL/min. The FID temperature was set at 300 °C with a sampling rate of 8 ms. The helium makeup gas flow was 24.0 mL/min, and the air and hydrogen flows were 200 mL/min and 32 mL/min, respectively. All gases were of 99.999% purity and provided by Linde Gas (Graz, Austria).

To facilitate identification of VSCs previously reported in the literature for their importance to flavor in chocolate (e.g., DMDS and DMTS), a standard mix of sulfur compounds containing dimethyl sulfide (DMS), dimethyl disulfide (DMDS), dimethyl trisulfide (DMTS), 3-(methylthio)propanal (= methional), and 2-methyl-3-(methyldithio)furan was analyzed in a similar manner as the chocolate samples. Only DMDS and DMTS were successfully identified in the cocoa samples based on their match with authentic standards. For retention index (RI) calculation [36], a n-alkane standard mix (C8–C20; Sigma-Aldrich) was analyzed in the same way as the samples—the FID signal was used to calculate the RIs.

### 4.3. Data Analysis

Comprehensive chromatography data was analyzed by the GCxGC analytical software program ChromSquare (v. 2.4, Shimadzu Europa GmbH, Duisburg, Germany) to semi-quantitate and to calculate the retention index (RI) in the first dimension for the 20 most abundant VSCs in the 27 cocoa liquor samples. Compound areas with the same RIs (+/− 5 units) were manually combined in the software to obtain the peak areas.

We estimated that we would be able to detect a minimum of 20 VSCs consistently in any given sample, so to apply the same semi-quantitation across all samples, we limited our quantitation to the top 20 most abundant VSCs in each sample. This is a rather conservative estimation that was made to minimize the risk of overestimating the number of VSCs present across all samples, but it is worth noting that more roasted samples in particular appeared to exceed this cut-off limit easily (see results).

Twenty-six unidentified VSCs were classified based on the retention times (RTs) of the peak area in both dimensions. Classifications were informed by a scatterplot modeling the RTs of each VSC peak in both dimensions to visualize the approximate location in which VSCs appeared. VSCs that appeared in less than three samples were excluded from further analysis.

For DMDS and DMTS, surface regression modeling was used to determine and visualize the impact of the quantitative experimental variables, roasting time and roasting temperature, and the qualitative variable, cacao origin. Details are described in [9,26], but in short, the full model, including all linear, interaction, and quadratic terms of the experimental factors (roasting time, roasting temperature, cacao origin), was fit and reduced to the most parsimonious model using a Bayesian information criterion (BIC). All statistical analysis took place in RStudio (Redmond, WA. USA) v. 2021.09.2, build 382 (“Ghost Orchid”), running R (Vienna, Austria) version 3.6.2. The package ggplot2 (v.3.3.6; [37]) was used for contour plots, the olsrr package (v.0.5.3; [38]) was used for all regression modelling, and the heatmaps were created with the ComplexHeatmap package (v.2.15.1; [39,40]).

## Figures and Tables

**Table 1:** Mean retention index (RI_1D_) ± standard deviation on the first dimension and the mean retention time (RT_2D_) ± standard deviation on the second dimension for the 28 detected VSCs. Unidentified compounds are labeled as CpdX, with X increasing in number with increasing RI on the first dimension.

Compound	RI_1D_ [-]	RT_2D_ [min]	Compound	RI_1D_ [-]	RT_2D_ [min]
DMDS	743 ± 1.5	1.98 ± 0.05	Cpd13	1091 ± 0.0	2.59 ± 0.02
DMTS	976 ± 0.0	3.33 ± 0.03	Cpd14	1092 ± 0.0	3.85 ± 0.03
Cpd1	688 ± 0.0	1.58 ± 0.02	Cpd15	1107 ± 0.0	3.29 ± 0.01
Cpd2	714 ± 1.2	2.33 ± 0.05	Cpd16	1131 ± 0.0	3.20. ± 0.03
Cpd3	910 ± 0.0	3.49 ± 0.05	Cpd17	1134 ± 0.0	4.12± 0.03
Cpd4	926 ± 3.4	4.71 ± 0.15	Cpd18	1181 ± 0.0	3.70 ± 0.03
Cpd5	1007 ± 0.0	3.30 ± 0.01	Cpd19	1243 ± 0.8	5.06 ± 0.06
Cpd6	1010 ± 0.0	4.28 ± 0.03	Cpd20	1246 ± 0.0	4.12 ± 0.03
Cpd7	1034 ± 0.0	3.02 ± 0.01	Cpd21	1253 ± 0.0	3.73 ± 0.03
Cpd8	1039 ± 4.0	3.64 ± 0.08	Cpd22	1253 ± 0.0	4.86 ± 0.04
Cpd9	1066 ± 1.5	5.01 ± 0.07	Cpd23	1267 ± 0.0	4.13 ± 0.03
Cpd10	1079 ± 0.0	3.23 ± 0.04	Cpd24	1292 ± 3.5	5.12 ± 0.06
Cpd11	1080 ± 0.8	4.28 ± 0.02	Cpd25	1310 ± 3.5	4.77 ± 0.06
Cpd12	1088 ± 0.0	3.36 ± 0.03	Cpd26	1398 ± 0.0	4.91 ± 0.05

**Table 2:** Model coefficients ± standard errors for the dimethyl disulfide (DMDS) and dimethyl trisulfide (DMTS) regression models. Coefficient estimates show changes in relative concentration. Significant model terms (*p* < 0.05) are indicated by an asterix (*).

Model Term	DMDS	DMTS
**(Intercept)**	−0.127 ± 0.122	−0.548 ± 0.096 *
**Origin: Madagascar**	−0.103 ± 0.154	-
**Origin: Peru**	−0.160 ± 0.154	-
**Time**	0.210 ± 0.061 *	0.281 ± 0.082 *
**Temp**	1.45 ± 0.094 *	0.938 ± 0.073 *
**Temp^2^**	0.685 ± 0.083 *	0.569 ± 0.071 *
**Time^2^**	−0.238 ± 0.068 *	-
**Time × Temp**	0.204 ± 0.060 *	-
**Origin: Madagascar × Temp**	−0.731 ± 0.126 *	-
**Origin: Peru × Temp**	−0.909 ± 0.126 *	-
**Origin: Madagascar × Temp^2^**	−0.418 ± 0.106 *	-
**Origin: Peru × Temp^2^**	−0.389 ± 0.106 *	-
**Degrees of freedom (df)**	15	23
**Residual Standard Error**	0.246	0.351
**Multiple R^2^**	0.965	0.891
**Adjusted R^2^**	0.939	0.877
**F-statistic**	37.6	62.7
***p***	6.85 × 10^−9^	3.18 × 10^−11^

**Table 3:** List of the 27 samples analyzed by GCxGC-SCD, based on a randomized modified I-optimal experimental design with a duplicated center point. Samples are numbered according to their roasting order (see also [9,26]).

No.	Time (min)	Temperature (°C)	Origin	No.	Time (min)	Temperature (°C)	Origin
12	0	24	Ghana	2/13	40	114	Ghana
23			Madagascar	14/20			Madagascar
5			Peru	1/11			Peru
25	55	64	Ghana	22	80	135	Ghana
16			Madagascar	26			Madagascar
10			Peru	21			Peru
7	80	84	Ghana	18	54	151	Ghana
24			Madagascar	3			Madagascar
4			Peru	9			Peru
19	11	105	Ghana	6	20	171	Ghana
17			Madagascar	8			Madagascar
15			Peru	27			Peru

## Data Availability

The data presented in this article is available on request from the corresponding authors.

## Sample Availability

Samples of the compounds are not available from the authors.
